# COVID-19 Advanced Care

**DOI:** 10.3390/jpm11111082

**Published:** 2021-10-25

**Authors:** Evangelia Fouka, Ioannis Kalomenidis, Niki Gianniou, Sofia Gida, Paschalis Steiropoulos

**Affiliations:** 1 Pulmonary Department, Aristotle University of Thessaloniki, G. Papanikolaou Hospital, 57010 Thessaloniki, Greece; 2 1st Department of Critical Care and Pulmonary Medicine, Evaggelismos Hospital, National and Kapodistrian University of Athens, 10679 Athens, Greece; ikalom@med.uoa.gr (I.K.); nikigianniou@yahoo.com (N.G.); 3 Intensive Care Unit, General Hospital of Trikala, 42100 Trikala, Greece; sophia.gida@gmail.com; 4 Department of Respiratory Medicine, Medical School, Democritus University of Thrace, 67100 Alexandroupolis, Greece; steiropoulos@yahoo.com

**Keywords:** SARS-CoV-2, severe acute respiratory syndrome, antiviral agents, systemic corticosteroids, monoclonal antibodies, passive immune therapies, anticoagulation, antibiotics

## Abstract

The coronavirus disease 2019 (COVID-19) pandemic, related to the novel severe acute respiratory syndrome coronavirus 2 (SARS-CoV-2), has caused a worldwide sudden and substantial burden in public health due to an enormous increase in hospitalizations for pneumonia with the multiorgan disease. Treatment for individuals with COVID-19 includes best practices for supportive management of acute hypoxic respiratory failure. Emerging data indicate that dexamethasone therapy reduces 28-day mortality in patients requiring supplemental oxygen compared with usual care, and ongoing trials are testing the efficacy of antiviral therapies, immune modulators and anticoagulants in the prevention of disease progression and complications, while monoclonal antibodies and hyperimmune globulin may provide additional preventive strategies. Consensus suggestions can standardize care, thereby improving outcomes and facilitating future research. This review discusses current evidence regarding the pharmacotherapy of COVID-19.

## 1. Introduction

At the end of 2019, a novel coronavirus was identified as the cause of a cluster of pneumonia cases in Wuhan, a city in the Hubei Province of China and shortly spread throughout China, followed by an increasing number of cases in other countries throughout the world [1]. In February 2020, the World Health Organization (WHO) designated the disease COVID-19, which stands for coronavirus disease 2019, while the virus that causes COVID-19 was designated as severe acute respiratory syndrome coronavirus 2 (SARS-CoV-2) [2].

The natural history of the COVID-19 infection can be divided into three main phases. Each phase of the disease has different symptoms that range from asymptomatic or mild disease to acute respiratory distress syndrome (ARDS) with multiorgan failure and shock. The early infection phase, involving viral replication, is characterized by mild or absent symptoms, while in the second pulmonary phase, which is driven by the host immune response, there is a predominance of respiratory symptoms, with hypoxemia, cough, shortness of breath and pneumonia, along with thrombotic events (especially in patients with comorbidities such as arterial hypertension, diabetes, chronic heart and respiratory disease and obesity), that may lead to acute hypoxemic respiratory failure (AHRF), multiorgan failure and shock [3]. In some cases, there is a third phase that leads to a rapid increase in inflammatory cytokines, such as ferritin, C-reactive protein, IL-1β, IL-1Ra, TNF-a, as well as IL-6, IL-10, IL-18 and IFN-γ, an uncontrolled inflammatory response known as cytokine storm syndrome that takes place after inflammatory cascade activation [4]. This hyperinflammation phase is a marker of the illness severity and increased mortality and may lead to rapid deterioration, with ARDS and multiorgan failure and need for intubation and ICU admission [5].

Until now, most viral infections tended to be treated with supportive care, and in some cases, antiviral therapy is used. As therapeutic interventions seem mandatory, at least in severe and critical COVID-19, in this study, we will review and discuss the supportive care along with specific, advanced approaches based on severity profile. For hospitalized patients, the standard of care mainly consists of adequate hydration, oxygen therapy, antibiotic use and thrombotic prophylaxis, accompanied by vital sign observation. Antivirals, along with immunomodulatory treatments, are more likely to be effective in the second and third phases of the disease [6]. Based on the latest literature, we will furthermore review and discuss these advanced and specific COVID-19 therapies.

## 2. Anti-Viral Agents

Several previously published studies have indicated that remdesivir, a viral RNA-dependent RNA polymerase inhibitor, may show some effectiveness against SARS-CoV-1, MERS-CoV and SARS-CoV-2 [7,8]. In the early ACTT1 trial [9], 1062 patients underwent randomization, 541 of whom were assigned to remdesivir and 521 to placebo, respectively. In that trial, reductions in recovery time and length of hospital stay (the primary outcomes of the study) were shown, from 15 to 10 days (rate ratio for recovery 1.29, 95% CI 1.12–1.49; *p* < 0.001) and from a median of 17 days to 12 days, respectively, while other secondary endpoints also showed positive benefits. However, no benefit on clinical recovery was observed in patients who received remdesivir at an advanced stage of the disease (symptoms >10 days) or in those who entered the study when they were already on mechanical ventilation or extracorporeal membrane oxygenation (recovery rate ratio 0.98, 95% CI 0.70–1.36). Goldman et al., in a SIMPLE-Severe study [10], demonstrated similar clinical improvement (≥2 points, in a 7-point ordinal scale) by day 14 with remdesivir given for 5 days versus 10 days in patients with severe COVID-19: 65% of patients in the 5-day group improved their clinical status versus 54% in the 10-day group (*p* = 0.14 for the baseline-adjusted difference between arms). Similarly, in a SIMPLE-Moderate study (a three-arm randomized, controlled trial in 584 hospitalized patients with SARS-CoV-2 infection and radiographic infiltrates by imaging with SpO2 > 94% on room air), Spinner et al. showed that patients receiving remdesivir for 5 days were 65% more likely to have clinical improvement on a 7-point ordinal scale at day 11 versus patients receiving standard of care alone (OR: 1.65; 95% CI: 1.09–2.48; *p* = 0.02), while the 10-day scheme did not show superiority versus the standard of care (No OR reported; *p* = 0.18) [11]. In contrast, other trials, including the large SOLIDARITY trial [12], demonstrated no evidence of benefits on mortality (rate ratio of 0.95; 95% CI 0.81–1.11; *p* = 0.50) or other clinical outcomes, regardless of age or need for ventilatory support. 

An updated meta-analysis of existing trials, including ACTT1, SOLIDARITY and other additional trials performed by the SOLIDARITY group, also demonstrated no mortality benefit of remdesivir (RR 0.91, 95% CI 0.79–1.05) [13]. Interestingly, the results of an ACTT2 study suggest that if remdesivir is given in combination with the Janus kinase-inhibitor baricitinib, significant decreases in time to recovery are observed in hospitalized patients with SARS-CoV-2 [14]. Nevertheless, as the effectiveness of remdesivir, with or without additional therapies, is unclear, further large studies are required to confirm its uselessness against the standard of care, with respect to endpoints, such as clinical improvement, clinical deterioration and length of stay, as well as to identify eligible subgroups of patients who will benefit from therapy, based on the timing of administration and ventilatory support requirements. 

Lopinavir, an HIV type 1 aspartate protease inhibitor, combined with ritonavir, has been previously shown to reduce the risk of adverse clinical outcomes and decrease the viral load in patients with SARS, as compared to historical controls [15,16]. However, three randomized trials, RECOVERY [17], SOLIDARITY [12] and a randomized, open-label Chinese trial [18], showed no effect of lopinavir–ritonavir combination on mortality in SARS-CoV-2 patients, while no other benefits were evident for various endpoints, including time to clinical improvement, viral load, viral clearance, discharge from hospital within 28 days and the need for invasive mechanical ventilation. Adverse events, including serious ones, were not increased, despite the known adverse event profile and drug–drug interactions of the lopinavir–ritonavir combination. [19,20]. However, as the drug does not seem effective and may theoretically present a potential for patient harm, clear evidence of its efficiency would be required to recommend its use, justifying a strong recommendation against its use currently.

Danoprevir is a potent hepatitis C virus (HCV) protease (NS3/4A) inhibitor, which was approved in 2018 in China for the treatment of hepatitis C. A small (n = 11) open-label, single-arm study evaluated the effects of danoprevir, boosted by ritonavir, in moderate COVID-19 patients hospitalized for pneumonia without respiratory failure. The primary endpoint of the study was the rate of composite adverse outcomes (defined as SPO2 ≤ 93% without oxygen supplementation, PaO2/FiO2 ≤ 300 mm Hg or a respiratory rate ≥30 breaths/min without supplemental oxygen), while the efficacy was also evaluated. Danoprevir/ritonavir (100/100 mg/day, per os) was well tolerated in all patients, and no composite adverse outcomes occurred during the study. After the initiation of the treatment, the first negative reverse real-time PCR (RT-PCR) test was detected at a median of 2 days (range 1–8 days); an improvement in infiltrates in CT scans was demonstrated at a median of 3 days (range 2–4 days); all patients were discharged from the hospital after 4–12 days of therapy. However, as the study was limited by its small sample size, the results need to be confirmed by large randomized trials [21].

## 3. Antibiotics

In a Wuhan-based study reporting outcomes and treatment for 191 patients hospitalized for COVID-19 [22], 50% of the deaths were imputable to secondary bacterial infections. The reported incidence of potential respiratory bacterial co-infections upon admission was 3.5% in several cohort studies, while secondary bacterial infections during hospitalizations occurred up to 15% of patients [23]. A systematic review on bacterial and fungal co-infections in COVID-19 patients reported an overall percentage of 8% of bacterial infections at any time during hospitalization, with the most common pathogens reported being: *S. aureus*, *H. influenzae*, *S. pneumoniae* and *K. pneumoniae*, while *Mycoplasma* spp., *Enterobacter ales*, *A. baumannii* and *P. aeruginosa* have also been reported [24]. Most bacterial pneumonias detected early enough can be safely and effectively treated with antibiotics [25]. Thus, antibiotics appear to be a crucial defense against mortality in COVID-19 patients. Broad-spectrum antibiotics are being widely used in hospitalized patients with COVID-19 pneumonia and/or signs of bacterial co-infection, with the most common antibiotic classes prescribed being fluoroquinolones, followed by macrolides, β-lactam/β-lactamase inhibitors and cephalosporins [26]. However, as excessive antibiotic use drives the emergence of antibiotic-resistant bacteria and antimicrobial resistance is by now a burning issue, antibiotics should be used with caution in COVID-19 patients. In daily practice, it is difficult to distinguish viral from bacterial pneumonia. As to what should be done with antibiotic therapies in the COVID-19 era, several guidelines have been additionally published, such as those from the Netherlands, the UK (NICE guidelines) and South Africa, as well as experts’ recommendations [24,27,28,29]. 

There appears to be a consensus among these documents that in the presence of suspected bacterial co-infection, particularly in most severe cases, local and/or national guideline-concordant antibiotics should be commenced. The 2020 Surviving Sepsis Campaign guideline on COVID-19 recommended treating critically ill patients admitted to the ICU with empiric antibiotic therapy within 1 h while waiting for the test results. If all the cultures are negative after 48–72 h of incubation, it may be reasonable to discontinue antibiotics, whereas if the microbiological results indicate the presence of a bacterial co-infection, antibiotic treatment may be able to be narrowed depending on the findings and should be continued for 5–7 days [30]. The evidence based on bacterial infections in COVID-19 is currently limited; therefore, large, randomized, controlled trials on the epidemiology of bacterial infections and antibiotic use in COVID-19 are needed.

## 4. Systemic Corticosteroids

As excessive inflammation seems to play an important role in severe COVID-19, there is a strong scientific rationale for the use of anti-inflammatory treatments, particularly systemic corticosteroids, to down-regulate the aggravated inflammatory cascade [31,32].

The most consistent outcome of corticosteroids’ efficacy in COVID-19 has been reported in the Randomized Evaluation of COVID-19 therapy (RECOVERY) trial [33], which enrolled 6425 hospitalized patients with COVID-19, 2104 of whom were randomized to receive dexamethasone (6 mg per day for 10 days) plus the standard of care or the standard of care alone. A significant reduction in mortality was reported with dexamethasone for both groups overall (22.9% versus 25.7%; *p* < 0.001), as well as in patients receiving oxygen (23.3% versus 26.2%) or mechanical ventilation (29.3% versus 41.4%) at randomization. However, no clear effect on mortality was manifested for patients with no supplementary oxygen requirements (17.8% versus 14.0% in dexamethasone and standard of care, respectively), with a pooled odds ratio for all patient subgroups of 0.70 (95% CI 0.48–1.01) and a greater mortality benefit in response to the treatment with dexamethasone for patients with a longer duration of symptoms. Still, the trial showed a numerically shorter median duration of hospitalization (12 versus 13 days), a statistically significant decreased need for invasive mechanical ventilation (risk ratio 0.77; 95% CI, 0.62 to 0.95) and a greater probability of discharge alive in 28 days (rate ratio 1.10; 95% CI 1.03–1.17) with dexamethasone plus the standard of care versus the standard of care alone. Similarly, the CoDEX trial [34] evaluated 299 patients with moderate or severe COVID-19-related ARDS (CARDS), concluding that dexamethasone plus standard of care resulted in a statistically significant increase in the number of days alive and in days free of mechanical ventilation over 28 days versus standard of care alone. Moreover, Vilar et al. [35] showed a beneficial mortality effect of high dose dexamethasone compared to a placebo in a prospective, multicenter, randomized, controlled trial of 314 patients with persistent moderate/severe CARDS.

Two RCTs of hydrocortisone [36,37] showed numerical, although non-significant, trends towards reduced mortality in hospitalized patients with COVID-19. In the REMAP-CAP open-label study (n = 384), a 7-day fixed-dose or shock-dependent dosing course of hydrocortisone resulted in reductions in hazard ratios for hospital and ICU length of stay in patients with severe COVID-19 [36]. On the contrary, the CAPE COVID trial (n = 149) was prematurely stopped, as no difference in reducing treatment failure, manifested as death or persistent respiratory failure, was exhibited with low-dose hydrocortisone versus comparators [37]. 

In a parallel, a double-blind, placebo-controlled and randomized trial of methylprednisolone (0.5 mg/kg) compared with the placebo plus standard of care, numerical non-significant trends towards reduced mortality were observed, with no difference in hospitalization outcomes [38] On the contrary, in a single-blind randomized controlled clinical trial, significantly reduced mortality and time to hospital discharge or death were reported with methylprednisolone pulse administration (250 mg·day−1 intravenously for 3 days) plus the standard of care versus the standard of care alone (5.9% versus 42.9%, *p* < 0.001 and median 11.6 versus 17.6 days, *p* = 0.006, respectively). A significant reduction in the proportion of patients receiving oxygen was also shown after 3 days of treatment with methylprednisolone compared to before treatment (82.4% versus 100%; *p* = 0.025) [39].

In a systematic review and meta-analysis of pooled data from seven RCTs conducted by the WHO Rapid Evidence Appraisal for COVID-19 Therapies (REACT) working group [40], the efficacy of different corticosteroid schemes (dexamethasone, hydrocortisone or methylprednisolone, 678 patients) versus usual care or a placebo (1025 patients) was evaluated in patients with critical COVID-19. This meta-analysis revealed that the administration of various types and doses of systemic corticosteroids was associated with a lower 28-day all-cause mortality compared to usual care or placebo, with an odds ratio of 0.61 (95% CI 0.48–0.78; *p* < 0.001) for low-dose schemes and 0.83 (95% CI 0.53–1.29; *p* = 0.46) for high-dose schemes, with the same benefit in mortality with either dexamethasone or hydrocortisone, suggesting a class effect (OR 0.70, 95% CI 0.48–1.01; *p* = 0.053).

In line with the results of the REACT Group, the WHO guideline on drugs for COVID-19 provided a strong recommendation for systemic corticosteroids in patients with severe and critical COVID-19 and a weak or conditional recommendation against their use in patients with non-severe disease [41]. Although the REACT trial was not conducted to assess the optimal dose and duration of treatment with systemic corticosteroids, the WHO proposes a relatively low dose, equivalent to 6 mg of dexamethasone, based on the RECOVERY trial findings [33]. Similarly, the ERS living guideline for hospitalized patients with COVID-19 strongly recommends the use of corticosteroids in patients requiring supplementary oxygen or ventilatory support and strongly contends their use in patients not requiring supplementary oxygen [42]. 

Although published data from the RECOVERY and other trials did not show major safety signals to date, the adverse event profile of systemic corticosteroids is well known, and evolving concern arises with their widespread use in patients with severe COVID-19. Of interest, two studies [43,44] report a considerable incidence (14.1% and 19.4%, respectively) of COVID-19-associated pulmonary aspergillosis (CAPA) development in mechanically ventilated patients with a history of chronic respiratory disease constituting an independent risk factor for CAPA. 

In conclusion, corticosteroids have been shown to significantly reduce mortality in randomized trials, with significantly different benefits according to disease severity based on the requirement for oxygen or mechanical ventilation, justifying different recommendations for different subgroups of patients. Unanswered questions regarding corticosteroids include the optimal molecule, timing, dosing and scheme, as well as the duration of treatment. Although concerns about potential adverse events due to their use seem reasonable, the consistency of results from most trials is reassuring about their risk-benefit profile.

## 5. Anticoagulants

The incidence of thromboembolic events in COVID-19 appears to be considerably higher compared to other critically ill or ARDS patients or in other respiratory virus infections known to lead to a procoagulant state [45]. A report from the American Society of Hematology states that the prevalence of DVT ranges from 1.1% among not critically ill patients to 69% among ICU patients [46]. The exact mechanism that leads to COVID-19 coagulopathy remains unclear, but several pathways have been proposed, such as complement-mediated thrombogenesis, the cytokine storm leading to neutrophil recruitment and NETosis and pneumonia mediated hypoxia, that stimulates platelet and neutrophil adhesion to endothelial cells while suppressing tissue factor pathway inhibitor and fibrinolytic pathways [47,48]. Disease severity in COVID-19 is associated with the prolongation of the prothrombin time (PT), international normalized ratio (INR) and variably by a trend toward shortened activated partial thromboplastin time (aPTT) [49].

Based on these observations, several institutions have developed anticoagulation protocols for patients infected with SARS-CoV-2. For non-hospitalized patients with COVID-19, the CDC proposes that anticoagulants should not be initiated for VTE prevention, unless other indications for the therapy exist or the patient is participating in a clinical trial [50]. Regarding thrombotic prophylaxis in hospitalized COVID-19 patients, relative agreement exists in the recommendations of several national and international scientific societies: the CDC recommends LMWH or UFH; the WHO recommends prophylactic daily LMWHs or twice-daily subcutaneous unfractionated heparin (UFH); the American Society of Hematology (ASH) suggests LMWH over UFH unless the risk of bleeding outweighs the risk of thrombosis; the American College of Cardiology (ACC) recommends that 40 mg of Enoxaparin daily or a similar LMWH can be administered with consideration of SC heparin (5000 IU twice to three times per day) in patients with renal dysfunction [49]. If pharmacological prophylaxis is contraindicated, mechanical VTE prophylaxis should be considered in immobilized patients [49].

In the recently published preliminary International Society on Thrombosis and Haemostasis (ISTH) Guideline, the use of LMWH in all hospitalized COVID-19 patients is suggested unless contraindicated. In addition, ISTH gives the option of using Fondaparinux in case there is a patient history of heparin-induced thrombocytopenia [50]. Although there are no current data on extended prophylaxis for COVID-19, this approach may be beneficial for patients recovering from pulmonary manifestations of this infection, especially those who are less mobile [51]. When indicated, the guidelines recommend either enoxaparin or rivaroxaban for a duration of 14 to 45 days post-discharge [46].

According to CDC, anticoagulation is contraindicated in active hemorrhage or severe thrombocytopenia, while ASH recommends thromboprophylaxis even with abnormal coagulation tests in the absence of active bleeding and held only if platelet count <25 × 10^9^/L or fibrinogen <0.5 g/L. Most of the other major societal guidelines and recommendations suggest holding anticoagulation in actively bleeding or severely thrombocytopenic patients [52].

Regarding thrombosis treatment in COVID-19, the CDC, ACCP and ACF recommend therapeutic anticoagulation in cases where there is a thromboembolic event or a high suspicion of a thromboembolic event when imaging is not possible. ASH recommends therapeutic anticoagulation only for documented clinical indications (e.g., VTE, atrial fibrillation or mechanical valve). SCC-ISTH recommends that therapeutic anticoagulation should not be considered for primary prevention until randomized trials are available. It is mentioned that therapeutic anticoagulation could be considered in patients without confirmed VTE but who present respiratory deterioration or ARDS. For the treatment of VTE in hospitalized patients with COVID-19, most guidelines suggest parenteral anticoagulation, with a switch to DOAC (assuming no drug interactions) as the patient transitions to the outpatient setting, and while using UFH in therapeutic doses, monitoring anti-Xa levels rather than an aPTT is proposed, as prolonged aPTT with elevated levels of factor VIII and positive lupus anticoagulants are common.

Concerning the duration of therapeutic anticoagulation, ACF recommends at least a 3-month course for patients who start anticoagulation for a presumed provoked thrombus from the inflammatory state of CAC but did not have imaging available for confirmation. Similarly, the ACCP and SCC-ISTH recommend a minimum of 3 months of anticoagulation in those patients with confirmed PE or proximal DVT. The ISTH-IG, ASH, ACC and CDC do not mention any recommendations or suggestions regarding the duration of therapeutic anticoagulation [52].

## 6. Anti-Inflammatory Agents

Several non-randomized cohort studies indicated the efficacy of anakinra, an IL-1 receptor antagonist, in moderate to severe COVID-19 pneumonia [53,54,55,56,57,58]. However, there is an essential need for randomized trials to confirm the benefit of anakinra in patients with COVID-19 pneumonia, hypoxia, respiratory failure and signs of progression into the third phase of the disease, dominated by hyperinflammation and an uncontrolled inflammatory response [59].

The CORIMUNO-ANA-1 multicenter, open-label, randomized clinical trial studied the efficacy of anakinra in 116 adult patients hospitalized with mild to moderate COVID-19 pneumonia presenting a score of 5 on the WHO Clinical Progression Scale (WHO-CPS), requiring at least 3 L/min of oxygen by mask or nasal cannula but in no need of ventilation assistance [60]. Patients were randomized to receive either anakinra (300 mg intravenously twice a day for days 1–3, 100 mg twice for day 4 and 100 mg once a day on day 5) plus standard care or standard care alone. In this trial, anakinra did not improve its two co-primary outcomes, as 36% of patients in the anakinra group had a WHO-CPS score of more than 5 at day 4 versus 38% in the standard care group (ARD-2.5%. 90% CI −17.1 to 12.0), and 47% of patients in the anakinra and 51% of patients in the standard care died or needed ventilation at day 14 (HR 0.97; 90% CI 0.62 to 1.52). Furthermore, survival up to day 90, defined as a secondary outcome in this trial, showed no difference between the anakinra and standard of care arms (27% in both groups), while 46% of patients receiving anakinra had a serious adverse event versus 38% in the usual care group (*p* = 0.45). Waiting for the results of randomized trials, such as the SAVE-MORE [61] and the ANA-COVID-GEAS [62] trial, to date, there are no randomized trials that support the use of this immunomodulatory treatment.

Between 23 April 2020 and 24 Jan 2021, 4116 adults of 21,550 COVID-19 hospitalized patients enrolled in the RECOVERY trial were included in the assessment of tocilizumab, an IL-6 receptor antagonist, including 3385 (82%) patients receiving systemic corticosteroids if they presented hypoxemia (oxygen saturation < 92% on air or requiring oxygen therapy) and evidence of systemic inflammation (C-reactive protein > 75 mg/L). Patients were randomized to the usual standard of care alone versus the usual standard of care plus tocilizumab at an intravenous dose of 400 mg to 800 mg, while an additional dose of tocilizumab could be given depending on the presence of clinical improvement or not [33]. The tocilizumab group was associated with a 4% lower rate in the primary outcome of all-cause mortality within 28 days compared to the standard of care group (31% vs. 35%, rate ratio 0.85; 95% CI 0.76–0.94; *p* = 0.0028). The patients allocated to tocilizumab were also more likely to be discharged from the hospital within 28 days (57% vs. 50% rate ratio 1.22; 1.12–1.33; *p* < 0.0001). The benefits of tocilizumab in this trial were seen in all prespecified subgroups of patients, including those receiving systemic corticosteroids. However, among those not receiving invasive mechanical ventilation at baseline, patients allocated to tocilizumab were less likely to reach the composite endpoint of invasive mechanical ventilation or death (35% versus 42%; risk ratio 0·84; 95% CI 0·77–0·92; *p* < 0.0001). 

The REMAP-CAP study [63], a randomized, multifractional, adaptive, open-label, international trial, enrolled 895 critically ill patients with a severe COVID-19 infection within the first 24 h after the initiation of organ respiratory or cardiovascular organ support, defined as the use of invasive or non-invasive mechanical ventilation (including high-flow oxygen devices, with a ≥30 L per minute and a fraction of inspired oxygen ≥ 0.4) and as intravenous administration of any vasopressor or inotrope and received respiratory support, respectively. Patients were randomized to receive an IL-6 receptor antagonist (tocilizumab 8 mg/kg or sarilumab 400 mg) plus the standard of care or the standard of care; to be noticed, the standard of care included glucocorticoids in most patients (>80%). The REMAP-CAP trial evidenced a reduction in the in-hospital mortality in the group of patients that received an IL-6 receptor antagonist versus the group that was treated with the standard of care (27% for the IL-6 receptor antagonist group versus 36% for the control group; hazard ratio 1.61; 95% CI 1.25 to 2.08, *p* < 0.001), while the median number of organ support-free days up to day 21 was 10 days for tocilizumab and 11 days for sarilumab versus 0 days for the control group (median pooled odds ratio 1.65; 95% CI 1.27 to 2.14, *p* < 0.001). Regarding the secondary outcomes of the REMAP-CAP, such as the 90-day survival, time to the intensive care unit (ICU), time to hospital discharge and improvement in the WHO ordinal scale at day 14, they were all superior to the IL-6 receptor antagonists’ group. No safety issues were noticed regarding the use of the IL-6 receptor antagonists compared to the standard of care, with nine serious adverse events seen in the tocilizumab group (one secondary bacterial infection, five bleeding events, two cardiac events and one case of vision deterioration), no serious adverse events in the sarilumab group and eleven in the control group (four bleeding events and seven cases of thrombosis) [63].

In advance of the RECOVERY and the REMAP-CAP trial, six numerically inferior randomized, controlled trials that studied the use of tocilizumab in patients with severe COVID-19 pneumonia did not result in lower mortality rates or an improvement in the clinical status of the patients [64,65,66,67,68,69]. In the COVACTA trial [64], 452 adult patients with severe COVID-19 pneumonia were randomized to receive a single intravenous dose of tocilizumab (8 mg/kg) plus the standard of care or a placebo in addition to the standard of care, and, based on clinical improvement or not, a second intravenous dose of tocilizumab was administrated. The COVACTA trial did not show evidence of an improvement in the clinical status of the patients receiving tocilizumab versus the control group at day 28, with a median value for clinical status on a seven-category scale of 1.0 versus 2.0, respectively (in between-group difference: −1.0; 95% CI −2.5 to 0.0, *p* = 0.31). In addition, as part of the secondary outcomes, the COVACTA trial did not show evidence of a reduction in the mortality rate at day 28 for the tocilizumab group compared to the placebo group (19.7% versus 19.4%; 95% CI: −7.6 to 8.2, *p* = 0.94); however, a possible benefit in time until discharge from the hospital and duration of ICU stay have been shown for patients who received tocilizumab (20 days versus 28 days; Cox proportional-hazard ratio, 1.35; 95% CI, 1.02 to 1.79 and 9.8 days versus 15.5 days; 5.8 difference 95% CI, −15.0 to 2.9, respectively). Regarding the safety of tocilizumab in this trial, an important difference for adverse events and serious adverse events between the tocilizumab and the control group has not been demonstrated [64].

In the TOCIBRAS study, an open-label, randomized trial that included 438 adults hospitalized with severe or critical COVID-19 pneumonia was conducted. Patients were randomized to receive either tocilizumab plus the standard of care versus a placebo plus the standard of care [65]. Standard of care included hydroxychloroquine, azithromycin, corticosteroids and antibiotics but not remdesivir, as that antiviral agent was not available. The TOCIBRAS trial was prematurely interrupted because of increased mortality at day 15 in the tocilizumab group compared to the control group (17% versus 3%; OR 6.42; 95% CI 1.59–43.2). Tocilizumab therapy was also not associated with an improvement in mechanical ventilation or death at day 15 (28% versus 20%, 95% CI, 0.66–3.66, *p* = 0.32).

The EMPACTA trial (Evaluating Minority Patients with Actemra) enrolled 389 adult hospitalized patients with COVID-19 pneumonia who were not receiving mechanical ventilation [66]. After randomization, patients received tocilizumab (8 mg/kg, one or two doses intravenously) plus standard care versus placebo plus standard care in the control group. The standard of care included antivirals, limited use of glucocorticoids (≤1 mg/kg of methylprednisolone or equivalent) and supportive care. Death from any cause by day 28 occurred in 10.4% of the patients in the tocilizumab group versus 8.6% in the control group (weighted difference, 2 percentage points; 95% CI, −5.2–7.8); however, this trial managed to show a reduction in the likelihood of progression to mechanical ventilation and death by day 28 in patients receiving tocilizumab (12% 95% CI, 8.5–16.9 for the tocilizumab group compared with 19.3%, 95% CI, 13.3–27.4 for the placebo group; hazard ratio, 0.56; 95% CI, 0.33–0.97 *p* = 0.04).

The CORIMUNO-TOCI trial [67] studied the effect of tocilizumab in 130 adult patients hospitalized with moderate to severe COVID-19 pneumonia. Patients in this cohort-embedded, investigator-initiated open-label, Bayesian-randomized clinical trial were receiving at least 3 L/min of oxygen but were not receiving mechanical ventilation or were admitted to the ICU. Patients randomized to the tocilizumab group, received 8 mg/kg intravenously on day 1 and on day 3 if clinically indicated plus standard care, while the control group received standard care including antibiotics, antivirals, corticosteroids, vasopressors and anticoagulants. According to the WHO 10-point Clinical Progression Scale (WHO-CPS) that was used to evaluate the clinical improvement of patients, tocilizumab failed to prove an improvement in the clinical status of patients by not reducing the WHO-CPS ≤5 at day 4, which was part of the primary outcome of the trial. The CORIMUNO-TOCI study also did not show evidence of a difference in mortality at day 28 between the tocilizumab and control groups, with seven deaths in the tocilizumab group versus eight in the control standard of care group (adjusted HR, 0.92; 95% CI 0.33–2.53). However, regarding the results of the secondary outcomes of the trial, 12% fewer patients in the tocilizumab group needed invasive or non-invasive mechanical ventilation or died on day 14 than in the control group (24% vs. 36%; HR 0.58; 90% CI, 0.33–1.00). Fewer serious adverse events were observed in the tocilizumab than in the control group (27 versus 57), including serious bacterial infections (2 versus 11).

The BACC Bay randomized, double-blind, placebo-controlled trial [68] enrolled 243 adult patients and evaluated the efficacy of tocilizumab in patients with moderate COVID-19 pneumonia (body temperature >38 °C, pulmonary infiltrates and need for supplemental oxygen in order to maintain an oxygen saturation ≥ 92%). Patients were allocated to receive tocilizumab 800 mg intravenously plus the standard of care (including antiviral therapy, hydroxychloroquine and glucocorticoids) or the standard of care. In this trial, the early use of tocilizumab did not reduce the need for intubation or mortality by day 28 (11.2% of the patients in the tocilizumab group were intubated or died versus 10.6% in the standard of care group; hazard ratio 0.83 95% CI, 0.38–1.81, *p* = 0.64). 

The RCT-TCZ-COVID trial [69] was designed to evaluate the effect of tocilizumab versus standard care on clinical worsening in adult patients hospitalized with COVID-19 pneumonia and enrolled 126 patients with PaO_2_/FiO_2_ between 200 and 300 mmHg, fever and elevated C-reactive protein. The tocilizumab group received intravenously two doses of tocilizumab (8 mg/kg, 800 mg maximum followed by a second dose after 12 h) plus standard of care, and the control group received standard of care alone. Steroids were not included in the standard of care and were allowed only for patients who used them before hospitalization. This study was prematurely interrupted for futility, as no differences between the tocilizumab and the control group were demonstrated by day 14 in all primary outcomes (intubation, mortality, clinical worsening).

The COVINTOC trial, an open-label, multicenter, randomized, controlled phase 3 trial, was published after the results of the RECOVERY and the REMAP-CAP trials and enrolled 180 adult patients hospitalized with moderate to severe COVID-19 pneumonia. Patients were randomized to receive tocilizumab (6 mg/kg, max 480 mg, second dose after 12 h up to 7 days, based on clinical progression) plus standard care or standard care alone in the control group. The primary endpoint was the progression of COVID-19 pneumonia from moderate to severe and from severe to death up to day 14. There was no difference between the tocilizumab and the control group in the progression of COVID-19 pneumonia (9% versus 13%; −3.71 95% CI-18.23–11.19, *p* = 0.42), as well as in all the secondary endpoints such as an improvement in cytokine release syndrome by day 28, the need for mechanical ventilation, organ failure-free days and overall mortality. No safety problems were indicated in this trial for the use of tocilizumab [70].

A meta-analysis of ten RCTs using tocilizumab, nine of which reported primary outcome data (mortality), showed that tocilizumab may be associated with an improvement in mortality (24.4% vs. 29.0%; OR 0.87 [0.74–1.01]; *p* = 0.07; I2 = 10%; TSA adjusted CI 0.66–1.14) [71]. Based on the RECOVERY and REMAP-CAP studies, the NIH COVID-19 treatment guidelines recommend the use of tocilizumab (8 mg/kg up to 800 mg, single intravenous dose) in combination with dexamethasone (6 mg up to 10 days) in adult COVID-19 patients: (a) recently hospitalized within 3 days of admission, admitted to the ICU within the prior 24 h and requiring invasive mechanical ventilation, non-invasive mechanical ventilation or high-flow nasal cannula oxygen (HFNCO) (>0.4 FiO_2_/30 L/min oxygen flow) (BIIa); (b) recently hospitalized, not admitted to the ICU, with increasing oxygen needs requiring non-invasive ventilation or HFNCO and significantly increased markers of inflammation (CRP ≥ 75 mg/L) (BIIa) [72]. Similarly, also based on the results of the RECOVERY and the REMAP-CAP studies, the IDSA guidelines [73] conditionally suggest the use of tocilizumab in addition to the standard of care, rather than the standard of care alone, in adult patients hospitalized with progressive severe or critical COVID-19 pneumonia (conditional recommendation, low certainty of evidence). Waiting for the MARIPOSA trial [74], the RECOVERY and the REMAP-CAP trials provide us with some evidence of the benefits of using tocilizumab under some circumstances.

## 7. Passive Immune Therapies

Therapeutic products containing anti-SARS-CoV-2 neutralizing antibodies obtained from recovered patients (convalescent plasma, hyper-immune anti-SARS-CoV-2 globulin) or artificial ones (monoclonal antibodies, Mabs) have attracted increased interest since the beginning of the COVID-19 pandemic. 

In late August 2020, the US Federal Drug Administration (FDA) issued EUA for convalescent plasma (CP) use in hospitalized patients based on retrospective observations indicating that patients treated with plasma containing high levels of IgG anti-SARS-CoV-2 antibodies have increased survival than those who received low-titer plasma [75]. Meanwhile, CP use profoundly increased globally, and several studies permitted a more detailed view of its role in CP in COVID-19 treatment. Phase II studies provided conflicting findings, while denoting a clinical benefit when high-titer CP was administered at early COVID-19 stages [76,77]. Disappointingly, several small and one large [78,79,80,81,82,83] clinical trials demonstrated no benefit of CP for hospitalized COVID-19 patients. On the other hand, high-titer CP administered within 72 h from symptom onset in patients ≥75 years old with mild COVID-19 was found to prevent disease progression [84]. However, a meta-analysis of randomized trials mainly including inpatients (more than 10,000) proved no impact of CP in COVID-19 outcomes [85]. In addition, weak observational evidence indicating that CP may be beneficial for patients with impaired humoral immunity [86,87,88,89,90,91,92,93,94,95] requires appropriate clinical testing. Based on the above, in February 2021, the revised FDA EUA limited the authorization to high-titer CP only for the treatment of hospitalized COVID-19 patients early in the disease course with impaired humoral immunity [75]. Currently, the National Institute of Health (NIH) guidelines do not recommend the use of low-titer CP for hospitalized patients with COVID-19 of any stage and permit high-titer CP use only in hospitalized patients with evidence of impaired immunity or in the context of a clinical trial [96]. No positive/negative suggestion is made for outpatients, given the lack of solid data. 

Several manufacturers are developing neutralizing anti-SARS-CoV-2 monoclonal antibodies that target the S-protein. As of June 2021, two Mabs combinations, bamlanivimab/etesevimab and casirivimab/imdevimab (also called REGN-COV2), and the Mab sotrovimab have been issued an FDA EUA for outpatients with mild/moderate COVID-19 and an increased risk of progression to severe disease [97]. Similarly, the European Medicines Agency (EMA) recommended a marketing authorization for the use of bamlanivimab/etesevimab and casirivimab/imdevimab combinations [7] and for the use sotrovimab [8] or regdanvimab [98] in high-risk patients with early-stage COVID-19, while the FDA-issued EUA for the bamlanivimab monotherapy was recently revoked, mainly because of concerns about the resistance of the new SARS-CoV-2 variants [97]. It should be emphasized that these therapies are not currently recommended for the treatment of hospitalized patients with severe COVID-19 disease, though research is ongoing. However, a recent trial testing bamlanivimab in hospitalized patients demonstrated that the addition of REGEN-COV to usual care reduces 28-day mortality of inpatients without detectable anti-SARS-CoV-2 antibodies at the baseline compared to usual care [99]. Even more interestingly, a study conducted during the autumn of 2020 found that bamlavinimab reduced the risk of infection by SARS-CoV-2 among residents and staff in skilled nursing and assisted-living facilities with positive cases, paving the way for the evaluation of Mabs as preventive tools [100]. 

It should be emphasized that, despite authorization, the clinical benefit of anti-SARS-CoV-2 Mabs is poorly defined. The initial evaluation of the Mabs was focused on their effects on the clearance of the virus from the upper airway, with the primary outcome being the differential change of the viral load from baseline through day 7–11, between treatment and control patients, as assessed by quantitative reverse transcriptase–polymerase chain reaction testing of nasopharyngeal swab samples [101]. In some studies [99,100,101], Mabs significantly accelerated the natural decline of the viral load, and it was thus anticipated that they potentially disrupt disease progression and may prevent health care facility visits, hospitalization and death. In the same line of evidence, unpublished data submitted to the FDA for EUA showed that sotrovimab significantly reduces the risk of hospitalization (1% vs. 7%) of outpatients that do not require oxygen therapy with an increased risk of progression [102]. However, such outcomes constituted secondary endpoints in these studies and the relevant observations, albeit encouraging, were of marginal importance, highlighting the fact that treating every patient with mild/moderate disease would be unrealistic and futile. Therefore, real-life data obtained from the increasing number of patients treated with Mabs are anticipated, and clinical trials with clinically relevant primary endpoints are required to reach certain conclusions. 

Apart from the uncertainty on their real clinical impact, other possible limitations of Mabs should be considered. A drawback lies in the fact that they are currently administered as an intravenous infection, and for this reason, the value of subcutaneous or intramuscular injections of Mabs is being investigated. On the other hand, while Mabs appears to be safe, hypersensitivity and infusion-related reactions may occur [101], meaning that they should be administered under medical observation. In addition, the emergence of resistance to the currently approved Mabs is an important concern, inherent to every type of antibody-based therapy. Attempting to address this issue, the FDA released information about in vitro resistance of the most widely circulating variants [97]. Variants carrying the E484K substitution (Brazil, South Africa and New York origin) demonstrated substantially increased in vitro resistance against bamlanivimab, and even though it is not clear whether in vitro susceptibility findings correlate with clinically important resistance, such observations caused FDA to revoke its initially issued EUA. 

As of June 2021, Mab combinations, especially REGN-COV2, appear to be active in the currently circulating variants. The NIH was the first organization to publish a clinical guideline that includes the use of bamlanivimab/etesevimab or casirivimab/imdevimab or sotrovimab for outpatients with mild/moderate COVID-19 and a high risk of clinical progression. According to the guideline, treatment should be started as soon as possible after diagnosis, within 10 days of symptom onset, and should not be given to hospitalized patients with severe COVID-19 [102].

Hyperimmune anti-COVID-19 IVIG was shown to be safe and potentially effective in a phase I/II trial [103]. In April 2021, the results of the Inpatient Treatment with an Anti-Coronavirus Immunoglobulin (ITAC) RCT Phase III trial were announced [104]. This trial randomized nearly 600 patients hospitalized for COVID-19, with symptoms for up to 12 days without life-threatening organ dysfunction or end-organ failure, to polyclonal SARS-CoV-2 hyperimmune IVIG plus standard treatment or standard treatment alone, and it was completely negative. 

## 8. Conclusions

Although the treatment of viral respiratory infections has traditionally been mostly supportive, the COVID-19 pandemic profoundly disrupted human activities and claimed millions of lives worldwide, thus forcing an unprecedented effort of health scientists, international organizations and pharmaceutical companies to develop novel, disease-specific therapies. According to the current model of COVID-19 pathobiology and in agreement with early clinical observations, it seems likely that antiviral agents, including antibody-based therapies, are more effective when administered early in the disease course, and they are anticipated to prevent the progression to severe/critical disease, which will lead to reductions in the need of hospitalization and mortality. During more progressed stages of the disease, when lung and systemic inflammation are driving the clinical course of COVID-19, anti-inflammatory and immuno-modulating agents might be required. Research aiming to discover more effective agents and the proper patient population to treat with them is underway.

## Data Availability

Not applicable.

## References

[1] Coronavirus Disease (COVID-19) Situation Reports. https://www.who.int/emergencies/diseases/novel-coronavirus-2019/situation-reports.

[2] WHO Director-General’s Opening Remarks at the Media Briefing on COVID-19—11 March 2020. https://www.who.int/director-general/speeches/detail/who-director-general-s-opening-remarks-at-the-media-briefing-on-covid-19---11-march-2020.

[3] The Natural History, Pathobiology, and Clinical Manifestations of SARS-CoV-2 Infections. https://www.ncbi.nlm.nih.gov/pmc/articles/PMC7373339.

[4] Leisman D.E., Ronner L., Pinotti R., Taylor M.D., Sinha P., Calfee C.S., Hirayama A.V., Mastroiani F., Turtle C.J., Harhay M.O. (2020). Cytokine elevation in severe and critical COVID-19: A rapid systematic review, meta-analysis, and comparison with other inflammatory syndromes. Lancet Respir. Med..

[5] Shi Y., Wang Y., Shao C., Huang J., Gan J., Huang X., Bucci E., Piacentini M., Ippolito G., Melino G. (2020). COVID-19 infection: The perspectives on immune responses. Cell Death Differ..

[6] Romagnoli S., Peris A., De Gaudio A.R., Geppetti P. (2020). SARS-CoV-2 and COVID-19: From the bench to the bedside. Physiol. Rev..

[7] Hrabovszki G. Treatments and Vaccines for COVID-19. https://www.ema.europa.eu/en/human-regulatory/overview/public-health-threats/coronavirus-disease-covid-19/treatments-vaccines-covid-19.

[8] Pinho A.C. EMA Issues Advice on use of Sotrovimab (VIR-7831) for Treating COVID-19. https://www.ema.europa.eu/en/news/ema-issues-advice-use-sotrovimab-vir-7831-treating-covid-19.

[9] Beigel J.H., Tomashek K.M., Dodd L.E., Mehta A.K., Zingman B.S., Kalil A.C., Hohmann E., Chu H.Y., Luetkemeyer A., Kline S. (2020). Remdesivir for the treatment of Covid-19—Final Report. N. Engl. J. Med..

[10] Goldman J.D., Lye D.C.B., Hui D.S., Marks K.M., Bruno R., Montejano R., Spinner C.D., Galli M., Ahn M.-Y., Nahass R.G. (2020). Remdesivir for 5 or 10 days in patients with severe Covid-19. N. Engl. J. Med..

[11] Spinner C.D., Gottlieb R.L., Criner G.J., Arribas López J.R., Cattelan A.M., Soriano Viladomiu A., Ogbuagu O., Malhotra P., Mullane K.M., Castagna A. (2020). Effect of Remdesivir vs standard care on clinical status at 11 days in patients with moderate COVID-19: A randomized clinical trial. JAMA.

[12] Pan H., Peto R., Henao-Restrepo A.-M., Preziosi M.-P., Sathiyamoorthy V., Abdool Karim Q., Alejandria M.M., Hernández García C., Kieny M.-P., WHO Solidarity Trial Consortium (2021). Repurposed antiviral drugs for covid-19—Interim WHO solidarity trial results. N. Engl. J. Med..

[13] Singh S., Khera D., Chugh A., Khera P.S., Chugh V.K. (2021). Efficacy and safety of remdesivir in COVID-19 caused by SARS-CoV-2: A systematic review and meta-analysis. BMJ Open.

[14] Kalil A.C., Patterson T.F., Mehta A.K., Tomashek K.M., Wolfe C.R., Ghazaryan V., Marconi V.C., Ruiz-Palacios G.M., Hsieh L., Kline S. (2021). Baricitinib plus remdesivir for hospitalized adults with Covid-19. N. Engl. J. Med..

[15] Chu C.M., Cheng V.C.C., Hung I.F.N., Wong M.M.L., Chan K.H., Chan K.S., Kao R.Y.T., Poon L.L.M., Wong C.L.P., Guan Y. (2004). Role of lopinavir/ritonavir in the treatment of SARS: Initial virological and clinical findings. Thorax.

[16] Yan D., Liu X.-Y., Zhu Y., Huang L., Dan B., Zhang G., Gao Y. (2020). Factors associated with prolonged viral shedding and impact of lopinavir/ritonavir treatment in hospitalised non-critically ill patients with SARS-CoV-2 infection. Eur. Respir. J..

[17] RECOVERY Collaborative Group (2020). Lopinavir-ritonavir in patients admitted to hospital with COVID-19 (RECOVERY): A randomised, controlled, open-label, platform trial. Lancet.

[18] Cao B., Wang Y., Wen D., Liu W., Wang J., Fan G., Ruan L., Song B., Cai Y., Wei M. (2020). A trial of lopinavir-ritonavir in adults hospitalized with severe covid-19. N. Engl. J. Med..

[19] Alhumaid S., Mutair A.A., Alawi Z.A., Alhmeed N., Zaidi A.R.Z., Tobaiqy M. (2020). Efficacy and Safety of lopinavir/ritonavir for treatment of COVID-19: A systematic review and meta-analysis. Trop. Med. Infect. Dis..

[20] Gutiérrez-Abejón E., Tamayo E., Martín-García D., Álvarez F.J., Herrera-Gómez F. (2020). Clinical profile, treatment and predictors during the first covid-19 wave: A population-based registry analysis from castile and leon hospitals. Int. J. Environ. Res. Public Health.

[21] Chen H., Zhang Z., Wang L., Huang Z., Gong F., Li X., Chen Y., Wu J.J. (2020). First clinical study using HCV protease inhibitor danoprevir to treat COVID-19 patients. Medicine.

[22] Zhou F., Yu T., Du R., Fan G., Liu Y., Liu Z., Xiang J., Wang Y., Song B., Gu X. (2020). Clinical course and risk factors for mortality of adult inpatients with COVID-19 in Wuhan, China: A retrospective cohort study. Lancet.

[23] Langford B.J., So M., Raybardhan S., Leung V., Westwood D., MacFadden D.R., Soucy J.-P.R., Daneman N. (2020). Bacterial co-infection and secondary infection in patients with COVID-19: A living rapid review and meta-analysis. Clin. Microbiol. Infect..

[24] Sieswerda E., De Boer M.G.J., Bonten M.M.J., Boersma W.G., Jonkers R.E., Aleva R.M., Kullberg B.-J., Schouten J.A., Van De Garde E.M.W., Verheij T.J. (2021). Recommendations for antibacterial therapy in adults with COVID-19—An evidence based guideline. Clin. Microbiol. Infect..

[25] Beović B., Doušak M., Ferreira-Coimbra J., Nadrah K., Rubulotta F., Belliato M., Berger-Estilita J., Ayoade F., Rello J., Erdem H. (2020). Antibiotic use in patients with COVID-19: A “snapshot” Infectious Diseases International Research Initiative (ID-IRI) survey. J. Antimicrob. Chemother..

[26] Langford B.J., So M., Raybardhan S., Leung V., Soucy J.-P.R., Westwood D., Daneman N., MacFadden D.R. (2021). Antibiotic prescribing in patients with COVID-19: Rapid review and meta-analysis. Clin. Microbiol. Infect..

[27] Metlay J.P., Waterer G.W. (2020). Treatment of community-acquired pneumonia during the coronavirus disease 2019 (COVID-19) pandemic. Ann. Intern. Med..

[28] COVID-19 Rapid Guideline: Managing Suspected or Confirmed Pneumonia in Adults in the Community|Guidance|NICE. https://www.nice.org.uk/guidance/ng165.

[29] Wu C.-P., Adhi F., Highland K. (2020). Recognition and management of respiratory co-infection and secondary bacterial pneumonia in patients with COVID-19. Clevel. Clin. J. Med..

[30] Alhazzani W., Møller M.H., Arabi Y.M., Loeb M., Gong M.N., Fan E., Oczkowski S., Levy M.M., Derde L., Dzierba A. (2020). Surviving sepsis campaign: Guidelines on the management of critically ill adults with Coronavirus Disease 2019 (COVID-19). Intensive Care Med..

[31] Yang J.-W., Yang L., Luo R.-G., Xu J.-F. (2020). Corticosteroid administration for viral pneumonia: COVID-19 and beyond. Clin. Microbiol. Infect..

[32] Xu Z., Shi L., Wang Y., Zhang J., Huang L., Zhang C., Liu S., Zhao P., Liu H., Zhu L. (2020). Pathological findings of COVID-19 associated with acute respiratory distress syndrome. Lancet Respir. Med..

[33] Horby P., Lim W.S., Emberson J.R., Mafham M., Bell J.L., Linsell L., Staplin N., Brightling C., Ustianowski A., RECOVERY Collaborative Group (2021). Dexamethasone in hospitalized patients with covid-19. N. Engl. J. Med..

[34] Tomazini B.M., Maia I.S., Cavalcanti A.B., Berwanger O., Rosa R.G., Veiga V.C., Avezum A., Lopes R.D., Bueno F.R., Silva M.V.A.O. (2020). Effect of dexamethasone on days alive and ventilator-free in patients with moderate or severe acute respiratory distress syndrome and covid-19: The CoDEX randomized clinical trial. JAMA.

[35] Villar J., Ferrando C., Martínez D., Ambrós A., Muñoz T., Soler J.A., Aguilar G., Alba F., González-Higueras E., Conesa L.A. (2020). Dexamethasone treatment for the acute respiratory distress syndrome: A multicentre, randomised controlled trial. Lancet Respir. Med..

[36] Angus D.C., Derde L., Al-Beidh F., Annane D., Arabi Y., Beane A., Van Bentum-Puijk W., Berry L., Bhimani Z., Bonten M. (2020). Effect of hydrocortisone on mortality and organ support in patients with severe covid-19: The REMAP-CAP COVID-19 corticosteroid domain randomized clinical trial. JAMA.

[37] Dequin P.-F., Heming N., Meziani F., Plantefève G., Voiriot G., Badié J., François B., Aubron C., Ricard J.-D., Ehrmann S. (2020). Effect of hydrocortisone on 21-day mortality or respiratory support among critically ill patients with covid-19: A randomized clinical trial. JAMA.

[38] Jeronimo C.M.P., Farias M.E.L., Val F.F.A., Sampaio V.S., Alexandre M.A.A., Melo G.C., Safe I.P., Borba M.G.S., Abreu-Netto R.L., Maciel A.B.S. (2020). Methylprednisolone as adjunctive therapy for patients hospitalized with covid-19 (Metcovid): A randomised, double-blind, phase iib, placebo-controlled trial. Clin. Infect. Dis..

[39] Edalatifard M., Akhtari M., Salehi M., Naderi Z., Jamshidi A., Mostafaei S., Najafizadeh S.R., Farhadi E., Jalili N., Esfahani M. (2020). Intravenous methylprednisolone pulse as a treatment for hospitalised severe COVID-19 patients: Results from a randomised controlled clinical trial. Eur. Respir. J..

[40] Sterne J.A.C., Murthy S., Diaz J.V., Slutsky A.S., Villar J., Angus D.C., Annane D., Azevedo L.C.P., Berwanger O., WHO Rapid Evidence Appraisal for COVID-19 Therapies (REACT) Working Group (2020). Association between administration of systemic corticosteroids and mortality among critically ill patients with covid-19: A meta-analysis. JAMA.

[41] Rochwerg B., Agarwal A., Siemieniuk R.A., Agoritsas T., Lamontagne F., Askie L., Lytvyn L., Leo Y.-S., Macdonald H., Zeng L. (2020). A living WHO guideline on drugs for covid-19. BMJ.

[42] Chalmers J.D., Crichton M.L., Goeminne P.C., Cao B., Humbert M., Shteinberg M., Antoniou K.M., Ulrik C.S., Parks H., Wang C. (2021). Management of hospitalised adults with coronavirus disease 2019 (COVID-19): A European Respiratory Society living guideline. Eur. Respir. J..

[43] Van Arkel A.L.E., Rijpstra T.A., Belderbos H.N.A., Van Wijngaarden P., Verweij P.E., Bentvelsen R.G. (2020). COVID-19-associated pulmonary aspergillosis. Am. J. Respir. Crit. Care Med..

[44] White P.L., Dhillon R., Cordey A., Hughes H., Faggian F., Soni S., Pandey M., Whitaker H., May A., Morgan M. (2020). A national strategy to diagnose COVID-19 associated invasive fungal disease in the ICU. Clin. Infect. Dis..

[45] Miesbach W., Makris M. (2020). COVID-19: Coagulopathy, risk of thrombosis, and the rationale for anticoagulation. Clin. Appl. Thromb. Hemost..

[46] Bikdeli B., Madhavan M.V., Jimenez D., Chuich T., Dreyfus I., Driggin E., Nigoghossian C.D., Ageno W., Madjid M., Guo Y. (2020). COVID-19 and thrombotic or thromboembolic disease: Implications for prevention, antithrombotic therapy, and follow-up. J. Am. Coll. Cardiol..

[47] Salabei J.K., Fishman T.J., Asnake Z.T., Ali A., Iyer U.G. (2021). COVID-19 coagulopathy: Current knowledge and guidelines on anticoagulation. Heart Lung.

[48] Kaptein F.H.J., Stals M.A.M., Huisman M.V., Klok F.A. (2021). Prophylaxis and treatment of COVID-19 related venous thromboembolism. Postgrad. Med..

[49] Gómez-Mesa J.E., Galindo-Coral S., Montes M.C., Muñoz Martin A.J. (2021). Thrombosis and coagulopathy in COVID-19. Curr. Probl. Cardiol..

[50] Ali M.A.M., Spinler S.A. (2021). COVID-19 and thrombosis: From bench to bedside. Trends Cardiovasc. Med..

[51] McBane R.D., Torres Roldan V.D., Niven A.S., Pruthi R.K., Franco P.M., Linderbaum J.A., Casanegra A.I., Oyen L.J., Houghton D.E., Marshall A.L. (2020). Anticoagulation in COVID-19: A systematic review, meta-analysis, and rapid guidance from mayo clinic. Mayo Clin. Proc..

[52] Flaczyk A., Rosovsky R.P., Reed C.T., Bankhead-Kendall B.K., Bittner E.A., Chang M.G. (2020). Comparison of published guidelines for management of coagulopathy and thrombosis in critically ill patients with COVID 19: Implications for clinical practice and future investigations. Crit. Care.

[53] Cavalli G., De Luca G., Campochiaro C., Della-Torre E., Ripa M., Canetti D., Oltolini C., Castiglioni B., Tassan Din C., Boffini N. (2020). Interleukin-1 blockade with high-dose anakinra in patients with COVID-19, acute respiratory distress syndrome, and hyperinflammation: A retrospective cohort study. Lancet Rheumatol..

[54] Huet T., Beaussier H., Voisin O., Jouveshomme S., Dauriat G., Lazareth I., Sacco E., Naccache J.-M., Bézie Y., Laplanche S. (2020). Anakinra for severe forms of COVID-19: A cohort study. Lancet Rheumatol..

[55] Cauchois R., Koubi M., Delarbre D., Manet C., Carvelli J., Blasco V.B., Jean R., Fouche L., Bornet C., Pauly V. (2020). Early IL-1 receptor blockade in severe inflammatory respiratory failure complicating COVID-19. Proc. Natl. Acad. Sci. USA.

[56] Navarro-Millán I., Sattui S.E., Lakhanpal A., Zisa D., Siegel C.H., Crow M.K. (2020). Use of anakinra to prevent mechanical ventilation in severe covid-19: A case series. Arthritis Rheumatol..

[57] Bozzi G., Mangioni D., Minoia F., Aliberti S., Grasselli G., Barbetta L., Castelli V., Palomba E., Alagna L., Lombardi A. (2021). Anakinra combined with methylprednisolone in patients with severe COVID-19 pneumonia and hyperinflammation: An observational cohort study. J. Allergy Clin. Immunol..

[58] Balkhair A., Al-Zakwani I., Al Busaidi M., Al-Khirbash A., Al Mubaihsi S., BaTaher H., Al Aghbari J., Al Busaidi I., Al Kindi M., Baawain S. (2021). Anakinra in hospitalized patients with severe COVID-19 pneumonia requiring oxygen therapy: Results of a prospective, open-label, interventional study. Int. J. Infect. Dis..

[59] WHO Working Group on the Clinical Characterisation and Management of COVID-19 Infection (2020). A minimal common outcome measure set for COVID-19 clinical research. Lancet Infect. Dis..

[60] CORIMUNO-19 Collaborative Group (2021). Effect of anakinra versus usual care in adults in hospital with COVID-19 and mild-to-moderate pneumonia (CORIMUNO-ANA-1): A randomised controlled trial. Lancet Respir. Med..

[61] suPAR-Guided Anakinra Treatment for Management of Severe Respiratory Failure by COVID-19 (SAVE-MORE). https://clinicaltrials.gov/ct2/show/NCT04680949.

[62] Clinical Trial of the Use of Anakinra in Cytokine Storm Syndrome Secondary to Covid-19 (ANA-COVID-GEAS) (ANA-COVID-GEAS). https://clinicaltrials.gov/ct2/show/NCT04443881.

[63] Gordon A.C., Mouncey P.R., Al-Beidh F., Rowan K.M., Nichol A.D., Arabi Y.M., Annane D., Beane A., Van Bentum-Puijk W., REMAP-CAP Investigators (2021). Interleukin-6 receptor antagonists in critically ill patients with covid-19. N. Engl. J. Med..

[64] Rosas I.O., Bräu N., Waters M., Go R.C., Hunter B.D., Bhagani S., Skiest D., Aziz M.S., Cooper N., Douglas I.S. (2021). Tocilizumab in hospitalized patients with severe covid-19 pneumonia. N. Engl. J. Med..

[65] Veiga V.C., Prats J.A.G.G., Farias D.L.C., Rosa R.G., Dourado L.K., Zampieri F.G., Machado F.R., Lopes R.D., Berwanger O., Azevedo L.C.P. (2021). Effect of tocilizumab on clinical outcomes at 15 days in patients with severe or critical coronavirus disease 2019: Randomised controlled trial. BMJ.

[66] Salama C., Han J., Yau L., Reiss W.G., Kramer B., Neidhart J.D., Criner G.J., Kaplan-Lewis E., Baden R., Pandit L. (2021). Tocilizumab in patients hospitalized with covid-19 pneumonia. N. Engl. J. Med..

[67] Hermine O., Mariette X., Tharaux P.-L., Resche-Rigon M., Porcher R., Ravaud P., CORIMUNO-19 Collaborative Group (2021). Effect of tocilizumab vs usual care in adults hospitalized with covid-19 and moderate or severe pneumonia: A randomized clinical trial. JAMA Int. Med..

[68] Stone J.H., Frigault M.J., Serling-Boyd N.J., Fernandes A.D., Harvey L., Foulkes A.S., Horick N.K., Healy B.C., Shah R., Bensaci A.M. (2020). Efficacy of tocilizumab in patients hospitalized with covid-19. N. Engl. J. Med..

[69] Salvarani C., Dolci G., Massari M., Merlo D.F., Cavuto S., Savoldi L., Bruzzi P., Boni F., Braglia L., Turrà C. (2021). Effect of tocilizumab vs standard care on clinical worsening in patients hospitalized with covid-19 pneumonia: A randomized clinical trial. JAMA Intern. Med..

[70] Soin A.S., Kumar K., Choudhary N.S., Sharma P., Mehta Y., Kataria S., Govil D., Deswal V., Chaudhry D., Singh P.K. (2021). Tocilizumab plus standard care versus standard care in patients in India with moderate to severe COVID-19-associated cytokine release syndrome (COVINTOC): An open-label, multicentre, randomised, controlled, phase 3 trial. Lancet Respir. Med..

[71] Snow T.A.C., Saleem N., Ambler G., Nastouli E., Singer M., Arulkumaran N. (2021). Tocilizumab in COVID-19: A meta-analysis, trial sequential analysis, and meta-regression of randomized-controlled trials. Intensive Care Med..

[72] Information on COVID-19 Treatment, Prevention and Research. https://www.covid19treatmentguidelines.nih.gov.

[73] IDSA Guidelines on the Treatment and Management of Patients with COVID-19. https://www.idsociety.org/COVID19guidelines.

[74] A Study to Investigate Intravenous Tocilizumab in Participants With Moderate to Severe COVID-19 Pneumonia (MARIPOSA). https://www.clinicaltrials.gov/ct2/show/NCT04363736.

[75] Commissioner O of the FDA Issues Emergency Use Authorization for Convalescent Plasma as Potential Promising COVID–19 Treatment, Another Achievement in Administration’s Fight Against Pandemic. https://www.fda.gov/news-events/press-announcements/fda-issues-emergency-use-authorization-convalescent-plasma-potential-promising-covid-19-treatment.

[76] Joyner M.J., Carter R.E., Senefeld J.W., Klassen S.A., Mills J.R., Johnson P.W., Theel E.S., Wiggins C.C., Bruno K.A., Klompas A.M. (2021). Convalescent plasma antibody levels and the risk of death from covid-19. N. Engl. J. Med..

[77] Simonovich V.A., Burgos Pratx L.D., Scibona P., Beruto M.V., Vallone M.G., Vázquez C., Savoy N., Giunta D.H., Pérez L.G., Sánchez M.D.L. (2021). A randomized trial of convalescent plasma in covid-19 severe pneumonia. N. Engl. J. Med..

[78] Li L., Zhang W., Hu Y., Tong X., Zheng S., Yang J., Kong Y., Ren L., Wei Q., Mei H. (2020). Effect of convalescent plasma therapy on time to clinical improvement in patients with severe and life-threatening covid-19: A randomized clinical trial. JAMA.

[79] Gharbharan A., Jordans C.C.E., Geurtsvankessel C., Den Hollander J.G., Karim F., Mollema F.P.N., Stalenhoef-Schukken J.E., Dofferhoff A., Ludwig I., Koster A. (2021). Effects of potent neutralizing antibodies from convalescent plasma in patients hospitalized for severe SARS-CoV-2 infection. Nat. Commun..

[80] Avendaño-Solà C., Ramos-Martínez A., Muñez-Rubio E., Ruiz-Antorán B., De Molina R.M., Torres F., Fernández-Cruz A., Callejas-Díaz A., Calderón J., Payares-Herrera C. (2021). A multicenter randomized open-label clinical trial for convalescent plasma in patients hospitalized with COVID-19 pneumonia. J. Clin. Invest..

[81] AlQahtani M., Abdulrahman A., Almadani A., Alali S.Y., Zamrooni A.M.A., Hejab A.H., Conroy R.M., Wasif P., Atkin S.L., Otoom S. (2021). Randomized controlled trial of convalescent plasma therapy against standard therapy in patients with severe COVID-19 disease. Sci. Rep..

[82] Biswas D., Maiti C., Talukder B., Azharuddin M., Saha S., Pandey S., Das A., Adhikari S.D., Ray Y., Sarkar B.S. (2021). A prospective study on COVID-19 convalescent plasma donor (CCP) recruitment strategies in a resource constrained blood centre. ISBT Sci. Ser..

[83] O’Donnell M.R., Grinsztejn B., Cummings M.J., Justman J., Lamb M.R., Eckhardt C.M., Philip N.M., Cheung Y.K., Gupta V., João E. (2021). A randomized, double-blind, controlled trial of convalescent plasma in adults with severe COVID-19. J. Clin. Investig..

[84] Libster R., Pérez Marc G., Wappner D., Coviello S., Bianchi A., Braem V., Esteban I., Caballero M.T., Wood C., Berrueta M. (2021). Early high-titer plasma therapy to prevent severe covid-19 in older adults. N. Engl. J. Med..

[85] Janiaud P., Axfors C., Schmitt A.M., Gloy V., Ebrahimi F., Hepprich M., Smith E.R., Haber N.A., Khanna N., Moher D. (2021). Association of convalescent plasma treatment with clinical outcomes in patients with covid-19: A systematic review and meta-analysis. JAMA.

[86] Ferrari S., Caprioli C., Weber A., Rambaldi A., Lussana F. (2021). Convalescent hyperimmune plasma for chemo-immunotherapy induced immunodeficiency in COVID-19 patients with hematological malignancies. Leuk. Lymphoma.

[87] Rahman F., Liu S.T.H., Taimur S., Jacobs S., Sullivan T., Dunn D., Baneman E., Fuller R., Aberg J.A., Bouvier N. (2020). Treatment with convalescent plasma in solid organ transplant recipients with COVID-19: Experience at large transplant center in New York City. Clin. Transpl..

[88] Mira E., Yarce O.A., Ortega C., Fernández S., Pascual N.M., Gómez C., Alvarez M.A., Molina I.J., Lama R., Santamaria M. (2020). Rapid recovery of a SARS-CoV-2-infected X-linked agammaglobulinemia patient after infusion of COVID-19 convalescent plasma. J. Allergy Clin. Immunol. Pract..

[89] Quinti I., Lougaris V., Milito C., Cinetto F., Pecoraro A., Mezzaroma I., Mastroianni C.M., Turriziani O., Bondioni M.P., Filippini M. (2020). A possible role for B cells in COVID-19? Lesson from patients with agammaglobulinemia. J. Allergy Clin. Immunol..

[90] Jin H., Reed J.C., Liu S.T.H., Ho H.-E., Lopes J.P., Ramsey N.B., Waqar O., Rahman F., Aberg J.A., Bouvier N.M. (2020). Three patients with X-linked agammaglobulinemia hospitalized for COVID-19 improved with convalescent plasma. J. Allergy Clin. Immunol. Pract..

[91] Balashov D., Trakhtman P., Livshits A., Kovalenko I., Tereshenko G., Solopova G., Petraikina E., Maschan A., Novichkova G. (2021). SARS-CoV-2 convalescent plasma therapy in pediatric patient after hematopoietic stem cell transplantation. Transfus. Apher. Sci..

[92] Senefeld J.W., Klassen S.A., Ford S.K., Senese K.A., Wiggins C.C., Bostrom B.C., Thompson M.A., Baker S.E., Nicholson W.T., Johnson P.W. (2021). Use of convalescent plasma in COVID-19 patients with immunosuppression. Transfusion.

[93] Clark E., Guilpain P., Filip I.L., Pansu N., Le Bihan C., Cartron G., Tchernonog E., Roubille C., Morquin D., Makinson A. (2020). Convalescent plasma for persisting COVID-19 following therapeutic lymphocyte depletion: A report of rapid recovery. Br. J. Haematol..

[94] Iaboni A., Wong N., Betschel S.D. (2021). A patient with X-Linked agammaglobulinemia and covid-19 infection treated with remdesivir and convalescent plasma. J. Clin. Immunol..

[95] Van Damme K.F.A., Tavernier S., Van Roy N., De Leeuw E., Declercq J., Bosteels C., Maes B., De Bruyne M., Bogaert D., Bosteels V. (2020). Case report: Convalescent plasma, a targeted therapy for patients with cvid and severe covid-19. Front. Immunol..

[96] Convalescent Plasma and Immune Globulins. https://www.covid19treatmentguidelines.nih.gov/therapies/anti-sars-cov-2-antibody-products/convalescent-plasma.

[97] COVID-19 Treatment Guidelines Panel Coronavirus Disease 2019 (COVID-19) Treatment Guidelines. National Institutes of Health. https://files.covid19treatmentguidelines.nih.gov/guidelines/covid19treatmentguidelines.pdf.

[98] Czarska-Thorley D. EMA Issues Advice on Use of Regdanvimab for Treating COVID-19. https://www.ema.europa.eu/en/news/ema-issues-advice-use-regdanvimab-treating-covid-19.

[99] Lundgren J.D., Grund B., Barkauskas C.E., Holland T.L., Gottlieb R.L., Sandkovsky U., Brown S.M., Knowlton K.U., Self W.H., ACTIV-3/TICO LY-CoV555 Study Group (2021). A neutralizing monoclonal antibody for hospitalized patients with covid-19. N. Engl. J. Med..

[100] Cohen M.S., Nirula A., Mulligan M.J., Novak R.M., Marovich M., Yen C., Stemer A., Mayer S.M., Wohl D., Brengle B. (2021). Effect of bamlanivimab vs placebo on incidence of covid-19 among residents and staff of skilled nursing and assisted living facilities: A randomized clinical trial. JAMA.

[101] Gottlieb R.L., Nirula A., Chen P., Boscia J., Heller B., Morris J., Huhn G., Cardona J., Mocherla B., Stosor V. (2021). Effect of bamlanivimab as monotherapy or in combination with etesevimab on viral load in patients with mild to moderate covid-19: A randomized clinical trial. JAMA.

[102] Anti-SARS-CoV-2 Monoclonal Antibodies. https://www.covid19treatmentguidelines.nih.gov/therapies/anti-sars-cov-2-antibody-products/anti-sars-cov-2-monoclonal-antibodies.

[103] Ali S., Uddin S.M., Shalim E., Sayeed M.A., Anjum F., Saleem F., Ali A., Ahmed I., Mushtaq T., Quraishy S. (2021). Hyperimmune anti-COVID-19 IVIG (C-IVIG) Treatment in Severe and Critical COVID-19 Patients: A phase I/II Randomized Control Trial. EClinicalMedicine.

[104] CoVIg-19 Plasma Alliance Announces Topline Results from NIH-Sponsored Clinical Trial of Investigational COVID-19 Hyperimmune Globulin Medicine. https://www.takeda.com/newsroom/newsreleases/2021/covig-19-plasma-alliance-announces-topline-results-from-nih-sponsored-clinical-trial-of-investigational-covid-19-hyperimmune-globulin-medicine.

